# Can you spot a liar? Deception, mindreading, and the case of autism spectrum disorder

**DOI:** 10.1002/aur.1962

**Published:** 2018-04-27

**Authors:** David M. Williams, Toby Nicholson, Catherine Grainger, Sophie E. Lind, Peter Carruthers

**Affiliations:** ^1^ School of Psychology University of Kent Canterbury Kent United Kingdom; ^2^ Psychology, Faculty of Natural Sciences University of Stirling Stirling Stirlingshire United Kingdom; ^3^ Department of Psychology City, University of London London United Kingdom; ^4^ Department of Philosophy University of Maryland College Park Maryland United States of America

**Keywords:** autism spectrum disorder, deception, lie detection, metacognition, mindreading, social cognition, theory of mind

## Abstract

Detection of deception is of fundamental importance for everyday social life and might require “mindreading” (the ability to represent others’ mental states). People with diminished mindreading, such as those with autism spectrum disorder (ASD), might be at risk of manipulation because of lie detection difficulties. In Experiment 1, performance among 216 neurotypical adults on a realistic lie detection paradigm was significantly negatively associated with number of ASD traits, but not with mindreading ability. Bayesian analyses complemented null hypothesis significance testing and suggested the data supported the alternative hypothesis in this key respect. Cross validation of results was achieved by randomly splitting the full sample into two subsamples of 108 and rerunning analyses. The association between lie detection and ASD traits held in both subsamples, showing the reliability of findings. In Experiment 2, lie detection was significantly impaired in 27 adults with a diagnosis of ASD relative to 27 matched comparison participants. Results suggest that people with ASD (or ASD traits) may be particularly vulnerable to manipulation and may benefit from lie detection training. ***Autism Res*** *2018, 11: 1129–1137*. © 2018 The Authors Autism Research published by International Society for Autism Research and Wiley Periodicals, Inc.

**Lay Summary:**

Detection of deception is of fundamental importance for everyday social life. People with diminished understanding of other minds, such as those with autism spectrum disorder (ASD), might be at risk of manipulation because of lie detection difficulties. We found that lie detection ability was related to how many ASD traits neurotypical people manifested and also was significantly diminished among adults with a full diagnosis of ASD.

## Introduction

The ability to detect when one is being deceived by others is of fundamental importance for everyday social life and difficulties detecting deception increase one's risk of being manipulated, with potentially serious consequences. Nonetheless, neurotypical adults tend to assume others are telling the truth (a default “truth‐bias”) and their ability to distinguish truths from lies in experimental situations is only just above chance, albeit statistically significantly so [54% across studies; Bond & DePaulo, 2008]. Moreover, there are few (if any) characteristics (e.g., age, sex, education) that appear reliably associated with lie detection ability [Aamodt & Custer, 2006]. These findings have led some to suggest that people vary in the extent to which they manifest behavioral indicators of honesty/deceit and that accurate inferences are possible only when judging people who provide consistent cues [e.g., Levine et al., 2011]. In other words, some honest individuals provide clear behavioral cues to indicate they are honest and some liars provide clear cues that they are dishonest. These relatively “transparent” individuals will be easier to make accurate judgments about than “nontransparent” liars, who hide the behavioral cues associated with lying, and nontransparent truth‐tellers who emit signs of dishonesty even though they are honest. Regardless, it may be that the ability to detect lies even in transparent individuals is underpinned by a particular set of psychological mechanisms or social experiences that have not yet been elucidated. Moreover, certain groups of people might have a diminished ability to detect lies, rendering them at particular risk of manipulation and social difficulties.

Intuitively, the abilities to lie and detect lies are an aspect of mindreading—the ability to explain and predict behavior in terms of underlying mental states (beliefs, desires, intentions, etc.). Lying to somebody involves an attempt to induce a false belief in them and, likewise, detection of someone else's lie requires interpretation of a person's behavior in terms of their intention to induce a false belief [Sip, Roepstorff, McGregor, & Frith, 2008]. Indeed, some have suggested that mindreading evolved precisely because it conferred an adaptive ability to manipulate others and detect when one is being manipulated without the need for physical conflict [e.g., Byrne & Whiten, 1997]. Although it is intuitive to consider lie detection as an aspect of mindreading, no studies have, to our knowledge, investigated the link between the two directly [although detection of suspicious behavior from auditory cues may be linked to mindreading ability; Brewer, Ying, Young, & Nah, 2018].

If there is a link between deception detection ability and mindreading ability, then individuals with diminished mindreading ability should also show impoverished lie detection skill. This is especially pertinent when considering the case of autism spectrum disorder (ASD). ASD is a neurodevelopmental disorder diagnosed on the basis of severe behavioral impairments in social‐communication and behavioral flexibility [American Psychiatric Association, 2013] that appear to be caused in part by an underlying deficit in mindreading [see Brunsdon & Happé, 2013; Jones et al., 2018]. People with ASD are thought to be particularly vulnerable to social manipulation, in part because of a difficulty in understanding lies. However, such a hypothesized difficulty with lie detection ability has never been investigated directly. Using cartoon‐type paradigms, studies have shown that individuals with ASD have difficulties understanding “double bluff,” in distinguishing lies from sarcasm/irony [Happé, 1994]. However, it is unclear how these indirect findings map on to true lie detection ability in realistic (nonhypothetical) situations. Moreover, even if lie detection ability turns out to be impoverished in ASD, it is not clear whether such a deficit is caused by the mindreading deficit that is well‐established in this disorder or by some other factor.

In the current study, two experiments investigated the underpinnings of lie detection ability and the extent to which it is impaired in ASD. In Experiment 1, we employed a realistic lie detection paradigm among 216 neurotypical adults. This task involved watching clips of university students being interviewed about whether they had cheated in an experiment that took place before the interview began. Half of the videos involved students who had cheated, but denied doing so, and half involved noncheating truth‐tellers. Ten of the videos showed transparent individuals, who gave relatively clear behavioral cues as to the veracity of their statements, and 10 showed nontransparent individuals whose behavioral cues were known to be difficult to interpret [based on ratings by judges in Levine, Shaw, & Shulman, 2010]. In Experiment 1, participants judged whether or not each individual was telling the truth. In addition, participants completed two widely employed cognitive‐experimental tests of mindreading ability, as well as a self‐report measure of ASD traits. We predicted that ASD traits would be negatively associated with overall accuracy of lie detection judgments and with accuracy of judgments of transparent individuals in particular (higher ASD traits = lower lie detection accuracy). In each case, we predicted that the significant association would be mediated by mindreading ability.

It is important to stress that many consider ASD to be a spectrum, given that (among other things) features of the disorder are distributed continuously throughout the general population with no clear separation between typical and clinical levels [e.g., Frazier et al., 2014], and family members of people with ASD frequently have elevated, but nonclinical, levels of ASD features relative to the population average [e.g., Piven et al., 1994]. Thus, studying individual differences in ASD traits and their relation to cognitive abilities in the general population has the potential to make an important contribution to our understanding of ASD itself. However, while there is continuity between ASD traits in the population and ASD features in diagnosed cases, there can still be qualitative differences in the cognitive mechanisms that underpin those traits in each population [e.g., Peterson, Wellman, & Liu, 2005]. Therefore, a full understanding requires the study of diagnosed cases, as well as traits in the general population. Thus, in Experiment 2, a group of adults with a full diagnosis of ASD, as well as age‐ and IQ‐matched comparison participants, completed the lie detection task, as well as measures ASD traits/feature severity. We predicted that participants with ASD would show significantly lower overall accuracy on the lie detection task. We further predicted that this diminution would be most pronounced when judging transparent individuals, given that even neurotypical comparison participants might show low accuracy when judging nontransparent individuals.

## Experiment 1: Method

### Participants

Two hundred and sixteen students (175 female) from the University of Kent (UK) took part in the experiment. The average age of participants was 19.38 years (*SD* = 2.35; range = 18–41) years. No participant had a history of ASD, according to self‐report. All participants gave informed consent and received course credit in partial fulfillment of their degree, for taking part in the study. The study (comprising Experiments 1 and 2) was ethically approved by School of Psychology Research Ethics Committee at the University of Kent.

### Materials and Procedures

#### Lie detection task

The 20 videos employed by Levine et al. [2011] and taken from Levine [2007−2011] were used in this study. Each video showed an adult being interviewed about their earlier participation in an experiment during which they had the opportunity to cheat by looking at an answer sheet while the experimenter was out of the room. An objective indicator of whether the individual had cheated was available, because (unknown to the individual) the individual's partner during the earlier experiment was actually a confederate. During the interview, individuals were asked a range of questions (e.g., about their enjoyment of trivia games). Crucially, at a particular point in the interview, the individuals were asked three questions about their behavior during the experiment. Two questions asked directly about cheating (“Did any cheating occur when the experimenter left the room?”; “Are you telling me the truth?”) and one was strategically designed to elicit behavioral cues of lie‐/truth‐telling (“What will your partner say when I ask her the same questions?”). Only the portion of the interviews that included the three critical questions were included in the current study. Half of the videos included individuals who had not cheated (truth‐tellers) and half included individuals who had cheated and who lied about this in the interview (liars). Importantly, the 20 videos employed in the current study were a subsample of 44 videos already rated by a large sample of judges in Levine et al. [2010]. Based on ratings of the 44 videos in Levine et al.'s [2010] study, the 20 videos employed in the current study were selected to contain a mixture of transparent and nontransparent individuals. This mixture was included to increase the range of responses and levels of accuracy among participants in the current study (in both experiments 1 and 2). In Experiment 1, we were particularly interested in the extent to which a person could read clear behavioral signs of deceit (in the condition involving transparent individuals) was associated with the number of ASD traits that they manifested (and, in Experiment 2, we were particularly interested in the extent to which individuals with a full diagnosis of ASD could detect these clear behavioral cues in the transparent condition).

Participants watched each video once and made a categorical judgment about whether the person being interviewed was lying or telling the truth about whether they cheated during the experiment. Videos were presented in a pseudo‐random order. Overall accuracy on the task was established using corrected hit rate [(proportion of truths correctly identified + proportion of lies correctly identified) − (proportion of truths incorrectly judged as lies + proportion of lies incorrectly judged as truths)]. A corrected hit rate (CHR) of zero would indicate chance‐level judgments on the task. CHR was also calculated separately for the transparent and nontransparent conditions. Finally, the proportion of truth judgments made by participants, independent of accuracy, was calculated. The higher the proportion, the greater the truth bias (i.e., tendency to believe that individuals in the videos were telling the truth).

#### Mindreading tasks

##### Reading the mind in the eyes task

The Reading the mind in the eyes (RMIE) task [Baron‐Cohen, Wheelwright, Hill, Raste, & Plumb, 2001] is a widely used measure of mindreading in clinical and nonclinical populations. Participants were presented with a series of 36 photographs of the eye‐region of the face. On each trial, participants were asked to pick one word from a selection of four to indicate what the person in the picture was thinking or feeling. Scores on the RMIE task range from a possible 0–36, with higher scores indicating better performance on the task.

##### Animations task

We employed a version of the “Animations” task as a second measure of mindreading [e.g., Abell, Happé, & Frith, 2000]. The task, which is based on Heider and Simmel [1944], required participants to describe interactions between a large red triangle and a small blue triangle, as portrayed in a series of silent video clips. Four clips were apt to invoke an explanation of the triangles’ behavior in terms of epistemic mental states, such as belief, intention, and deception. These clips comprise the “mentalizing” condition of the task and were employed in this study.

Each clip was presented to participants on a computer screen. After the clip was finished, participants described what had happened in the clip. An audio recording of participants’ responses was made for later transcription. Transcriptions were scored on a scale of 0–2 for accuracy (including reference to specific mental states), based on the criteria outlined in Abell et al. [2000]. Twenty percent of transcripts were also scored by two independent raters. Inter‐rater reliability was excellent according to Cicchetti's [1994] criteria (intra‐class correlations >.82).

A *Z* score was calculated for each mindreading task. The two *Z* scores were then averaged to form a composite mindreading score. The composite was used in bivariate and partial correlation analyses in order to reduce the number of statistical comparisons and maximize power. However, following an anonymous reviewer's suggestion, we also report post hoc correlations with RMIE and Animations separately.

#### Measure of ASD traits

##### Autism‐spectrum quotient

The Autism‐spectrum Quotient [AQ; Baron‐Cohen, Wheelwright, Skinner, Martin, & Clubley, 2001]. The AQ is used widely, and is a valid and reliable measure of ASD traits in people with a full diagnosis and in the general population. Participants read statements (e.g., “I find social situations easy”; “I find myself drawn more strongly to people than to things”) and decide the extent to which each statement applies to them, responding on a 4‐point Likert scale, ranging from “definitely agree” to “definitely disagree.” Scores range from 0 to 50, with higher scores indicating more ASD traits.^1^

### Statistical Power and Analysis

Details of power calculations can be found in Supporting Information. An increasingly used supplement to power analyses and null hypothesis significance testing in general is to calculate a Bayes factor for each key analysis. Bayesian analyses provide an estimation of the relative strength of a finding for one hypothesis over another (i.e., the alternative hypothesis over the null, or vice versa), which allows a more graded interpretation of the data than is possible using *P* values or effect sizes alone [e.g., Dienes, 2014; Rouder, Speckman, Sun, Morey, & Iverson, 2009]. According to Jeffreys’ [1961] criteria, Bayes factors (BF_10_) > 3 provide firm evidence for the alternative hypothesis (with values >10, >30, and >100 providing strong, very strong, and decisive evidence, respectively) and values under 1 provide evidence for the null (with values <0.33 providing firm evidence). BF_10_ values can be considered to reflect the likelihood that the alternative hypothesis is more likely to be true than the null hypothesis. Hence, a BF_10_ of 3 suggests the alternative hypothesis is three times more likely to be true than the null hypothesis. Bayesian analyses were conducted using JASP 0.8.1 [JASP Team, 2016].

## Experiment 1: Results

### Performance on the Lie Detection and Background Tasks

Means (*SD*) for performance on the experimental and background tasks are presented in Table 1. A repeated‐measures analysis of variance (ANOVA), with Condition (transparent/nontransparent) as the within‐participants variable, was conducted on accuracy data from the deception detection task. The effect of Condition was significant, reflecting significantly greater accuracy in the transparent condition than in the nontransparent condition, *F*(1, 215) = 1105.13, *P* < .001, $\eta_{p}^{2}=$ .84. One‐sample *t*‐tests showed that overall accuracy (i.e., CHR) and accuracy (CHR) in the transparent condition was significantly above chance, *t*s > 21.20, *p*s < .001, BF_10_s > 100. However, CHR in the nontransparent condition was significantly below chance, *t* = 7.54, *P* < .001 BF_10_ >100. Finally, participants showed a significant truth bias, *t* = 8.01, *P* < .001, BF_10_ > 100.^2^

**Table 1:** Mean (*SD*) Performance on Tasks in Experiment 1 (*N* = 216 Participants)

Variable	Mean (*SD*)
**Lie detection**	
CHR: overall	.28 (.19)
CHR: transparent condition	.71 (.27)
CHR: nontransparent condition	−.14 (.27)
Truth bias	.56 (.11)
**Background measures**	
AQ total	17.00 (6.66)
RMIE	25.24 (4.14)
Animations	4.19 (1.96)

### Association Analyses

Associations between each of the key dependent variables on the lie detection task and performance on each of the background measures are presented in Table 2. As predicted, AQ score was significantly negatively associated with overall accuracy (CHR) on the lie detection task. Moreover, AQ score was significantly negatively associated with accuracy of judgments in the transparent condition (as predicted). However, neither the size of the truth bias, nor accuracy of judgments in the nontransparent condition, correlated significantly with AQ. Importantly, none of the lie detection dependent variables was associated with the mindreading composite score. Note also that none of the lie detection dependent variables was associated significantly with either RMIE (all *r*s ≤ .02, all *p*s ≥ .73) or animations (all *r*s ≤ .12, all *p*s ≥ .09) mindreading tasks when they were analyzed individually.

**Table 2:** Bivariate Correlations in Experiment 1

	1	2	3	4	5	6
1. CHR: overall	–	.70***^b^	.71***^b^	.11	−.18**^a^	.04
2. CHR: transparent condition	–	–	.05	.04	−.26***^b^	.09
3. CHR: nontransparent condition	–	–	–	.11	<.01	−.04
4. Truth bias	–	–	–	–	−.10	.05
5. AQ total	–	–	–	–	–	−.13^*^
6. Mindreading composite	–	–	–	–	–	–

***P < .001; **P < .01; *P < .05.

BF_10_ > 3.

BF_10_ > 100.

AQ = autism‐spectrum quotient; CHR = corrected hit rate; RMIE = reading the mind in the eyes.

To investigate further the significant association between lie detection accuracy and AQ, two partial correlation analyses were conducted. The association between overall CHR and AQ remained significant after controlling for mindreading composite score, *r*_p_ = −.17, *P* = .01. Controlling for RMIE or animations task performance separately produced the same results as when the composite mindreading score was controlled (*p*s ≤ .01). Likewise, the association between CHR in the transparent condition and AQ remained significant after controlling for mindreading composite score, *r*_p_ = −.25, *P* < .001. Controlling for RMIE or animations task performance separately produced the same results as when the composite mindreading score was controlled (*p*s ≤ .001).

### Cross‐Validation of Results

Given that a number of authors have suggested that significant correlates of lie detection ability may not be replicable across studies [e.g., Aamodt & Custer, 2006; Bond & DePaulo, 2008], we assessed the reliability of the current findings by randomly splitting our sample into two groups of 108 participants and reanalyzing the data in each subsample.^3^

The association between CHR in the transparent condition and AQ after controlling for score on the mindreading composite measure was significant in both Subsample 1, *r*_p_ = −.23, *P* = .02, and Subsample 2, *r*_p_ = −.27, *P* = .005. The association between overall CHR and AQ after controlling for score on the mindreading composite measure was significant in Subsample 1, *r*_p_ = −.22, *P* = .02, but marginally nonsignificant in subsample 2 when reported one‐tailed, *r*_p_ = −.13, *P* = .08. Fisher's Z tests revealed that the difference in the size of these associations in the total sample, subsample 1, and subsample 2 were all nonsignificant (all *Z*s < 0.44, all *p*s > .67). Note that all results were substantively identical when analyses controlled for RMIE and Animations task performance separately (i.e., no result that was significant when controlling for the mindreading composite score became nonsignificant when controlling for RMIE and Animations task performance separately; vice versa, no result that was nonsignificant became significant).

## Experiment 2: Method

### Participants

Twenty‐seven adults with ASD and 27 neurotypical comparison adults took part. All participants completed the Wechsler Abbreviated Scale for Intelligence‐II [Wechsler, 1999], which provides verbal, performance, and full‐scale IQ scores. Participant characteristics and matching statistics are presented in Table 3. Participants in the ASD group had received verified diagnoses, according to conventional criteria [American Psychiatric Association, 2000; World Health Organisation, 1992]. No participant in either group reported current use of psychotropic medication or illegal recreational drugs, and none reported any history of neurological or psychiatric illness other than ASD.

**Table 3:** Participant Characteristics and Matching Statistics for Experiment 2

	ASD (*n* = 27; 20 male)	Comparison (*n* = 27; 17 male)	*t*	*P*	*d*
Age (years)	33.13 (13.64)	33.60 (11.83)	0.14	.89	0.04
VIQ	104.96 (11.08)	105.52 (7.97)	0.21	.83	0.05
PIQ	103.52 (14.19)	104.37 (11.20)	0.25	.81	0.07
FSIQ	104.44 (11.03)	105.67 (8.83)	0.45	.66	0.12
AQ total	30.04 (9.33)	16.30 (6.05)	6.42	<.001	1.75
ADOS	8.13 (4.94)	–	–	–	–

ADOS = autism diagnostic observation schedule; AQ = autism‐spectrum quotient; FSIQ = full scale IQ; PIQ = performance IQ; VIQ = verbal IQ.

### Materials and Procedures

Participants from each group completed the same versions of the lie detection task and AQ as participants completed in Experiment 1. In addition, participants with ASD completed the Autism Diagnostic Observation Schedule [ADOS; Lord et al., 2000], a detailed observational assessment of ASD features.

## Experiment 2: Results

### Performance on the Lie Detection and Background Tasks

**Figure 1:**
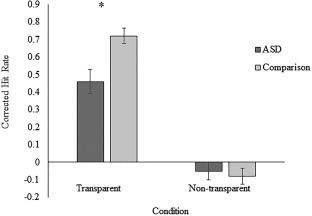
Mean performance on lie detection task in Experiment 2, in both the ASD and comparison group. Error bars represent one *SEM*. *P < .01.

A mixed ANOVA was conducted on lie detection CHR, with Group (ASD/comparison) as the between‐participants variable and Condition (transparent/nontransparent) as the within‐participants variable. This analysis revealed significant main effects of Condition, *F*(1, 52) = 174.72, *P* < .001, $\eta_{p}^{2}=$ .77, and Group, *F*(1, 52) = 3.94, *P* = .05, $\eta_{p}^{2}=$ .07, and a significant interaction between these variables, *F*(1, 52) = 8.48, *P* = .005, $\eta_{p}^{2}=$ .14. Figure 1 shows the breakdown of performance by ASD and comparison participants in each condition. CHR in the transparent condition was substantially and significantly lower among ASD participants (*M* = .46, *SD* = .36) than among comparison participants (*M* = .72, *SD* = .23), *t* = 3.16, *P* = .003, *d* = 0.86, BF_10_ = 27.86, albeit significantly above chance among both groups, all *t*s > 6.65, all *p*s < .001, all BF_10_s > 100. In contrast, CHR in the nontransparent condition was almost identical among ASD participants (*M* = −.05, *SD* = .27) and comparison participants (*M* = −.08, *SD* = .24), *t* = 0.42, *P* = .67, *d* = 0.11, BF_10_ = 0.30, and nonsignificantly below chance in both groups, *t*s > 6.65, all *p*s < .001, all BF_10_s < 0.33. In sum, participants with ASD showed a significant diminution of overall lie detection accuracy and this was particularly large in the transparent condition. Finally, both groups of participants showed a truth bias and there was no significant difference between participants with ASD (*M* = .55, *SD* = .15) and comparison participants (*M* = .57, *SD* = .12) in the size of the bias, *t* = 0.41, *P* = .69, *d* = 0.15, BF_10_ = 0.29.^3^

### Association Analyses

A series of bivariate correlation analyses were conducted to explore the association between lie detection ability and ASD traits/features. However, these were exploratory and not necessarily reliable given the small sample size in Experiment 2 relative to that in Experiment 1.

Among participant groups combined (*n* = 54), overall CHR was nonsignificantly associated with AQ (*r* = −.18, *P* = .20, BF_10_ = 0.69), although the correlation was almost identical in magnitude to that observed among participants in Experiment 1 (Table 2). These correlations remained nonsignificant when explored in each diagnostic group separately (*p*s > .67). In the ASD group, ADOS was nonsignificantly associated with overall CHR, *r* = −.03, *P* = .88, BF_10_ = 0.28.

Among participant groups combined, CHR in the transparent condition was associated significantly with AQ (*r* = −.28, *P* = .04, BF_10_ = 2.50). However, neither of these correlations was significant when explored in each diagnostic group separately (all *p*s > .89). In the ASD group, ADOS was nonsignificantly associated with CHR in the transparent condition (*r* = −.20, *P* = .35, BF_10_ = 0.63).

## General Discussion

In Experiment 1, overall CHR (i.e., overall lie detection accuracy) was significantly above chance, replicating previous studies. However, when looking at each condition separately, it became clear that only judgments in the transparent condition were significantly above chance. This was expected, given previous findings of low lie detection accuracy when judging nontransparent individuals [Levine et al., 2011]. Most importantly, and in keeping with predictions, lie detection accuracy was significantly negatively associated with number of ASD traits in Experiment 1. This was true for overall accuracy, as well as for accuracy in the transparent condition. Importantly, these results held in two subsamples (each comprising 108 participants) created by randomly splitting the total sample in half. Given that some have argued that reliable correlates of lie detection ability may never be found [see Bond & DePaulo, 2008], the replication of the link between lie detection and ASD traits in the current study is striking.

Arguably, the association between lie detection and ASD traits should not be surprising, given that such judgments are fundamentally social in nature and that ASD is at its core a disorder of social functioning and cognition. However, contrary to predictions, this association was not mediated by mindreading ability. Although there are some questions over the ecological validity of the animations and RMIE tasks [e.g., Cook, Brewer, Shah, & Bird, 2013], we included them as measures of mindreading in the current study for several reasons. For example, unlike other classic measures (e.g., false belief tasks), they are sensitive to mindreading impairments among intellectually high‐functioning individuals with ASD and to variation in mindreading skills among neurotypical individuals [e.g., Castelli et al., 2002; Lind et al., 2013]. The fact that deception detection ability was not associated with performance on either the animations or RMIE task (or a composite of performance across both tasks), suggests that lie detection ability is not related to mindreading ability directly. Rather, lie detection ability might develop as a function of the degree to which one engages with others socially, and attends to and learns from behavioral cues. As ASD traits increase, the tendency to engage in the kind of social interaction that would provide a means of learning about such behavioral cues is reduced. Although mindreading clearly contributes to social‐communication ability (better mindreaders are more socially skilled), the mere tendency to initiate social interaction may not depend on mindreading [Frith, Happé, & Siddons, 1994]. Therefore, a sufficient degree of social engagement, along with general‐purpose learning abilities, may be enough to learn the behavioral cues associated with transparent truths and lies.

Importantly, the results from Experiment 2 were striking and complemented those from Experiment 1. Adults with a diagnosis of ASD showed significantly diminished lie detection ability, relative to closely matched neurotypical participants. The impairment when judging videos of transparent individuals was associated with a large effect size, reflecting the fact that judgments by participants with ASD about transparent individuals were almost half as accurate as those made by comparison participants. This shows that even when people provide clear behavioral cues about their honesty or deceit, individuals with ASD nonetheless have significant difficulty making accurate judgments. For example, a clear verbal indicator of dishonesty is apparent in one of the videos, in which an individual claims not to have cheated when the interviewer asks “Did any cheating occur when the experimenter left the room?” but makes a Freudian slip and answers “no” to the follow‐up question, “Are you telling me the truth, right now?” before correcting himself and saying “I mean, yes”. In another of the videos, an individual responds, “I *guess* no” to the question “Did any cheating occur when the experimenter left the room?” We suggest that such behavioral cues would cause most neurotypical individuals to suspect deceit, but yet participants with ASD in the current study found it difficult to make such an inference when clear behavioral cues were available. Clearly, this difficulty renders individuals with a full diagnosis at risk of manipulation even by transparent individuals whose lies would be readily detectable by neurotypical individuals.

The underlying reasons for the observed lie detection difficulties in people with ASD are yet to be established. As argued above, they may be attributable to insufficient learning opportunities, which are the consequence of social impairments. Alternatively, there may be ASD‐specific cognitive differences that make deception detection more difficult. For example, the fact that lie detection inherently carries a high executive load (receivers must hold in mind and evaluate multiple cues, and consider counterfactual information) may mean that people with ASD—who often have executive difficulties—may be overloaded.

Irrespective of the underlying explanation for lie detection difficulties in ASD, it is important to consider whether training individuals with ASD to detect the behavioral indicators of lying (e.g., providing a vague or implausible account with few specific details; appearing ambivalent; assertions that lack of certainty/assertiveness) would be beneficial. Notably, such lie detection training has produced limited success in increasing discrimination accuracy among neurotypical adults [e.g., Frank & Feeley, 2003]. This may be because less‐than‐perfect lie detection accuracy among neurotypical individuals is not the result of a *lack of awareness* of the behavioral cues associated with truth‐telling and lying, but because the cues themselves are not consistently *strong* indicators [Hartwig & Bond, 2011]. In that case, instructing neurotypical individuals about behavioral indicators of lying that they are already aware of will not be fruitful. However, given that participants with ASD had such difficulties discriminating truths and lies told by even transparent individuals who displayed clear signs of their honesty or dishonesty, it seems likely that individuals with ASD are not fully aware of the cues that can be used to discriminate truth‐telling and lying. As such, providing explicit training about the nature of such cues might well be beneficial among people with this neurodevelopmental disorder. If such training was successful, it would represent a significant opportunity to enhance the lives of a group of people who, on the basis of our results and anecdotal reports, are clearly susceptible to exploitation.

## Footnotes

1 In addition to the AQ, participants also completed the Toronto Alexithymia Questionnaire (TAS‐20; Bagby, Parker, & Taylor, 1994). The TAS‐20 is a 20‐item self‐report measure of self‐awareness of one's emotional and physical states. The TAS was completed as part of a wider investigation of the relation between mindreading and self‐awareness, but was not central to the current study and so was not included in analyses.

2 There were no significant differences between males and females in terms of either level of accuracy or patterns of performance across conditions, all *p*s > .22, all $\eta_{p}^{2}<$ .007, all BF_10_s < 0.33.

3 There were no significant differences between the subsamples in characteristics (age, gender ratio), performance on the background tasks (RMIE, Animations, AQ), all *p*s > .29, all *d*s < 0.15, all BF_10_s < 0.25 or lie detection accuracy (all *p*s > .78, all $\eta_{p}^{2}<$ .001, all BF_10_s < 0.15).
